# EZH2 inhibition re-sensitizes multidrug resistant B-cell lymphomas to etoposide mediated apoptosis

**DOI:** 10.18632/oncoscience.288

**Published:** 2016-01-29

**Authors:** Matthew Smonskey, Elena Lasorsa, Spencer Rosario, Jason S. Kirk, Francisco J. Hernandez-Ilizaliturri, Leigh Ellis

**Affiliations:** ^1^ Department of Pharmacology and Therapeutics, Roswell Park Cancer Institute, Buffalo, NY, USA; ^2^ Department of Medicine, Roswell Park Cancer Institute, Buffalo, NY, USA; ^3^ Department of Molecular Pharmacology and Cancer Therapeutics, State University of New York at Buffalo, NY, USA

**Keywords:** lymphoma, EZH2, etoposide, therapy, epigenetics

## Abstract

Reactivation of apoptotic pathways is an attractive strategy for patients with treatment-resistant B-cell lymphoma. The tumor suppressor, p53 is central for apoptotic response to multiple DNA damaging agents used to treat aggressive B-cell lymphomas, including etoposide. It has been demonstrated that etoposide induced DNA damage and therapeutic efficacy is enhanced by combination with inhibitors of the histone methyltransferase, enhancer of zeste homolog 2 (EZH2). Further, EZH2 was identified to regulate cell fate decisions in response to DNA damage. Using B-cell lymphoma cell lines resistant to etoposide induced cell death; we show that p53 is dramatically down regulated and MDMX, a negative regulator of p53, is significantly up regulated. However, these cell lines remain responsive to etoposide mediated DNA damage and exhibit cell cycle inhibition and induction of senescence. Furthermore, chemical inhibition of EZH2 directs DNA damage to a predominant p53 dependent apoptotic response associated with loss of MDMX and BCL-X_L_. These data provide confirmation of EZH2 in determining cell fate following DNA damage and propose a novel therapeutic strategy for patients with aggressive treatment-resistant B-cell lymphoma.

## INTRODUCTION

The tumor suppressor *TP53* (p53) is inactivated or mutated in multiple cancers, including B-cell lymphoma [1]. P53 action in response to DNA damage most often occurs by the activation of genes involved in cell-cycle arrest, senescence, and apoptosis [2]. Genotoxic chemotherapy agents such as etoposide mediate therapeutic efficacy via p53-DNA damage signaling. Etoposide represents one of multiple genotoxic chemotherapy options used in conjunction with rituximab to treat aggressive B-cell lymphomas. Unfortunately, upon failure, further treatment options are limited; therefor a need for novel therapeutic directions is needed.

Failure to achieve clinical responses in patients using genotoxic chemotherapy in part maybe attributed by induction of senescence rather than apoptosis by p53. Multiple studies indicate that p53-mediated senescence impairs the apoptotic response to chemotherapy in various cancers [3-6], resulting in chemo-resistance and limited patient response to such therapies. More recently, the polycomb protein enhancer of zeste homolog 2 (EZH2) was demonstrated to regulate cancer cell fate in response to DNA damage [7]. EZH2 is a histone methyltransferase that is frequently overexpressed in various human cancers. Mechanistically, EZH2 catalyzes trimethylation of histone H3 lysine 27 (H3K27me3) resulting in gene suppression and its action is implicated in cell proliferation, apoptosis and senescence [8].

Previously, our investigations have demonstrated significant combinatorial effect of etoposide with EZH2 inhibition that resulted in increased cellular apoptosis associated with sustained DNA damage in models of lethal prostate cancer [9]. Also, independent studies confirmed the advantage of this combination in models of non-small cell lung cancer with *BRG1* or *EGFR* mutations [10].

This study investigated the p53 response of two independent multidrug resistant B-cell lymphoma models to etoposide. We demonstrate that etoposide treatment of multidrug resistant B-cell lymphomas results in p53 activation and DNA damage associated with induction of cell cycle arrest and senescence. Inhibition of EZH2 in multidrug resistant B-cell lymphomas directed etoposide mediated DNA damage response towards a p53 dependent apoptotic response associated with the loss of MDMX and BCL-XL expression. These data broaden the relevance and utility of etoposide and EZH2 inhibitor combination as a novel therapeutic strategy for aggressive B-cell lymphomas.

## RESULTS

### Multidrug resistant B-cell lymphomas exhibit reduced response to etoposide-mediated cell death with associated aberration in p53 and MDMX expression

**Figure 1:**
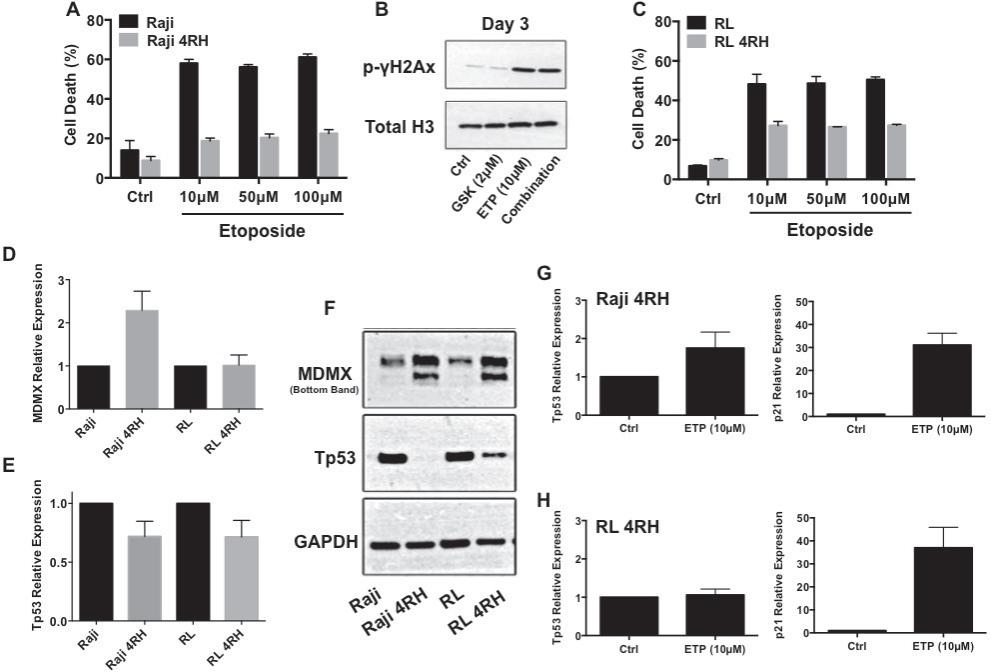
**A. Raji and Raji-4RH cell lines were treated with indicated concentrations of etoposide for 72 hours.** Cell death was determined by uptake of propidium iodide and quantitated by flow cytometry. **B.** Histone extracts from Raji-4RH cells were assessed for levels of p-γH2Ax by western blot. Total histone H3 was used as loading control. **C.** RL and RL-4RH cell lines were treated with indicated concentrations of etoposide for 72 hours. Cell death was determined by uptake of propidium iodide and quantitated by flow cytometry. **D-E.** Raji, Raji 4RH, RL and RL 4RH cell lines were assessed for baseline MDMX and Tp53 mRNA expression by real-time PCR. GAPDH was used as a reference housekeeper gene. **F.** Western blotting for baseline protein expression of MDMX and Tp53 was using assessed Raji, Raji 4RH, RL and RL 4RH whole cell lysates. GAPDH was used as loading control. **G-H.** Raji, Raji 4RH, RL and RL 4RH cell lines were treated with 10μM etoposide for 24 hours and assessed for Tp53 and p21 mRNA expression by real-time PCR. GAPDH was used as a reference housekeeper gene. Cell death and PCR experiments represent mean ±SEM of 3 independent experiments.

It was previously demonstrated that repeated exposure of B-cell lymphoma cell lines RL and Raji to rituximab induced a rituximab-resistant phenotype (RL-4RH and Raji-4RH) [11]. Surprisingly, RL-4RH and Raji-4RH cell lines showed significant resistance to multiple chemotherapy agents with distinct mechanisms of action. Of interest, was acquired resistance to the p53 dependent DNA damaging agent etoposide. Olejniczak et al [11] demonstrated that RL-4RH and Raji-4RH cell lines remained resistant to etoposide mediated apoptosis at 100μM. Treatment of parental and resistant RL and Raji cell lines with increasing concentrations of etoposide confirmed resistance to be dose independent (Figure 1A and 1C). Because of the observed resistance to etoposide mediated cell death, we investigated if etoposide still induced DNA damage. Treatment Raji 4RH cell lines with low dose etoposide (10μM) resulted in significant levels of DNA damage, which were also maintained when combined with the EZH2 inhibitor, GSK126 (Figure 1B). Etoposide mechanism of action is p53 dependent; therefore the status of p53 and regulators of p53 was of interest. Basal expression of p53 mRNA and protein was dramatically reduced in RL-4RH and Raji-4RH cell lines when compared to their respective parental lines. Further, an increase in MDMX, a negative regulator of p53 expression, mRNA was observed in Raji-4RH but not RL-4RH when compared to each parental, though significant increase in MDMX protein levels occurred in each resistant cell line (Figure 1D-1F). These data indicate that resistance to etoposide-mediated apoptosis is acquired in part by modulation of the MDMX-p53 signaling axis.

### Multidrug resistant B-cell lymphomas display p53-p21 response to etoposide associated with increases in G2M arrest and cellular senescence

**Figure 2:**
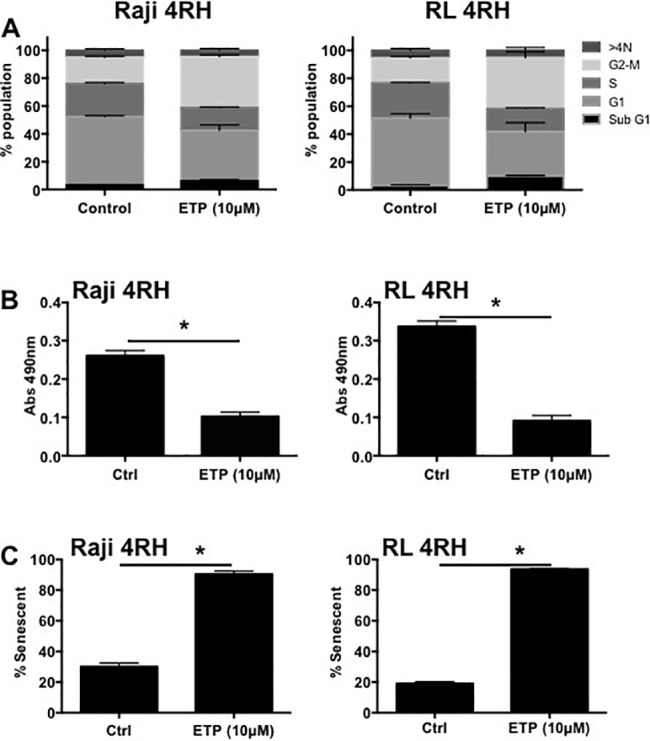
**Raji 4RH and RL 4RH cell lines were treated with 10μM etoposide for 72 hours and assessed for A.** cell cycle by flow cytometry, **B.** cell proliferation by MTS assay and **C.** senescence by detection of β-galactosidase activity. All experiments represent mean ±SEM of 3 independent experiments. *p<0.05.

While RL-4RH and Raji-4RH cell lines were resistant to etoposide-mediated apoptosis, it was unclear if p53 was still functional and if resistance to all p53 mediated mechanisms including cell cycle arrest and senescence were abrogated. Treatment for 72 hours with 10μM etoposide showed marked increase of p53 mRNA and the p53 target gene, p21 in Raji-4RH cells (Figure 1G). RL-4RH cells did not show marked increased of p53 mRNA, though p21 expression was still increased indicating potential p53 activity (Figure 1H). Further analysis revealed that following treatment RL-4RH and Raji-4RH cell lines resulted in marked increased cell cycle arrest in the G2M phase (Figure 2A), reduced cell proliferation (Figure 2B) and increased senescence (Figure 2C). Overall, these data demonstrate that p53 response to etoposide signaling is still active in RL-4RH and Raji-4RH cell lines.

### Targeting EZH2 results in cell death and loss of H3K27me3

**Figure 3:**
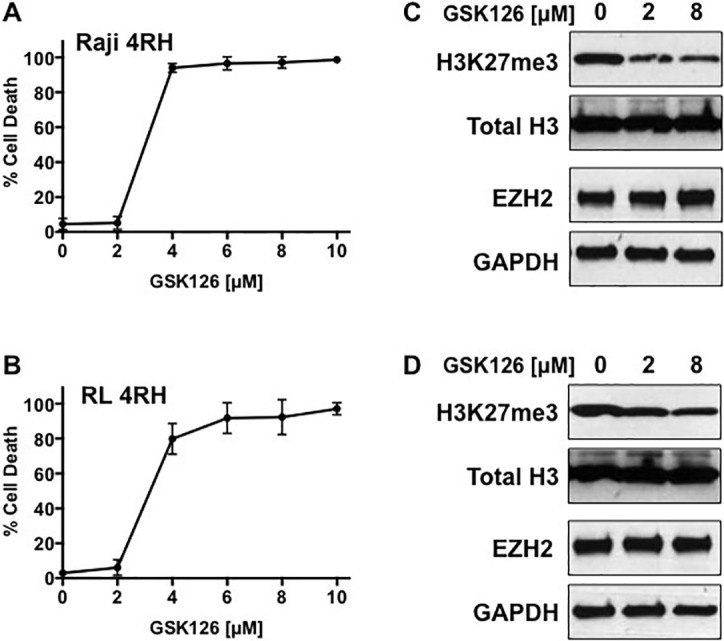
Raji 4RH and RL 4RH cell lines were treated with indicated concentrations of GSK126 for 14 days **A.** Cell death was determined by uptake of propidium iodide and quantitated by flow cytometry. **B.** Raji 4RH and RL 4RH cell lines were treated with indicated concentrations of GSK126 for 48 hours. Histone extractions and whole cell lysates were used to assess protein expression of H3K27me3 and EZH2 respectively by western blot. Total histone H3 and GAPDH were used as respective loading controls. Cell death experiments represent mean ±SEM of 3 independent experiments.

Recently, EZH2 has become a high priority therapeutic target in multiple cancers, including aggressive B-cell lymphomas. In addition, EZH2 is demonstrated to dictate cell fate in response to DNA damage and combination of etoposide with EZH2 inhibitors elicits potent anti-tumor activity [9, 10]. Treatment of RL-4RH and Raji-4RH cells with increasing concentrations of the EZH2 inhibitor, GSK126 [12], for 14 days showed similar loss of cell membrane integrity between both cell lines (Figure 3A-3B). Interestingly, inhibition of H3K27me3 occurred equally between a non-cytotoxic and cytotoxic concentration of GSK126 (2μM verse 8μM) without loss of EZH2 protein in both cell lines (Fig 3C-3D). These data demonstrate that multidrug resistant B-cell lymphomas maintain sensitivity to EZH2 inhibitors, and that GSK126 induced cytotoxicity is independent of EZH2 histone methyltransferase activity.

### Combination treatment by EZH2 inhibition and etoposide result in sustained p53 expression and DNA damage associated with inhibition of MDMX and BCL-X_L_ expression

**Figure 4:**
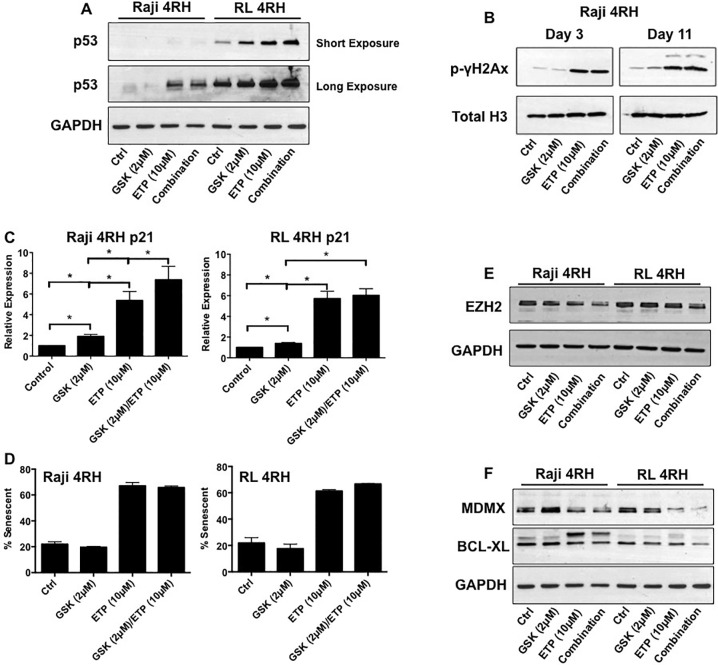
Raji 4RH and RL 4RH cell lines were treated as indicated for 11 days **A.** Western blot analysis was performed to assess p53 protein expression using whole cell lysates. GAPDH was used as a loading control. **B.** Western blot analysis was performed to assess p-γH2AX protein expression using histone extractions. Total histone H3 was used as a loading control. **C.** Real-time PCR was used to assess p21 mRNA expression. GAPDH was used as a reference housekeeper gene. **D.** Cellular senescence was assessed by detection of β-galactosidase activity. **E-F.** Western blot analysis was performed to assess EZH2, MDMX, and BCL-XL protein expression. GAPDH was used as a loading control. PCR experiments represent mean ±SEM of 3 independent experiments. *p<0.05.

Previously, our work and others have demonstrated the greater anti-tumor activity of etoposide combined with EZH2 inhibitors [9, 10]. It was therefor of high interest to examine the potential of this combination in these multidrug resistant B-cell lymphoma models. Figure 2 of our data indicated that p53 was still responsive to etoposide treatment through cell cycle arrest and increased senescence. With this, we further analyzed p53, EZH2 and MDMX response following long term (11 days) drug exposure. Analysis by western blot indicates that both etoposide single treatment and combination with GSK126 equally induces p53 protein expression (Figure 4A). Analysis of DNA damage in Raji-4RH cells by p-γH2AX expression following short and long-term drug treatments (day 3 verse day 11) demonstrate that both etoposide single treatment and combination induce similar levels of DNA damage that is equally maintained over time by both treatments (Figure 4B). In line with sustained p53 protein expression was increased expression of p21 mRNA. The cyclin dependent kinase inhibitor p21 is known to be a primary gene target and mediator of p53 checkpoint activation and senescence [13, 14]. Across both cell lines, GSK126 treatment significantly increases p21 mRNA, though does not activate cellular senescence (Figure 4C-4D). Etoposide treatment dramatically increases p21 mRNA, which is expected due the p53 response to etoposide. This significant increase of p21 mRNA also occurs with combination treatment (Figure 4C). Aligned with increased p21 expression, etoposide and combination treatment also maintained significant increases in senescence (Figure 4D) that was associated with loss of EZH2 protein expression when compared to control and GSK126 treated cells (Figure 4E). As noted in Figure 1, MDMX protein expression was dramatically up regulated in RL-4RH and Raji-4RH. Following 11 days of treatment, GSK126 treatment appeared to have little effect on MDMX protein expression. Etoposide alone did reduce MDMX levels, though combination treatment displayed greatest reduction of MDMX protein expression (Figure 4F). Further, it was observed that reduction of the anti-apoptotic protein; BCL-XL was reduced by combination treatment when compared to other treatment groups (Figure 4F). These data indicate that while long-term treatment retains p53-p21 senescence signaling, combination treatment results in increased inhibition of key proteins regulating epigenetic gene regulation, p53 signaling and apoptosis.

### Combination of etoposide with EZH2 inhibition increases apoptotic response in multidrug resistant B-cell lymphomas

**Figure 5:**
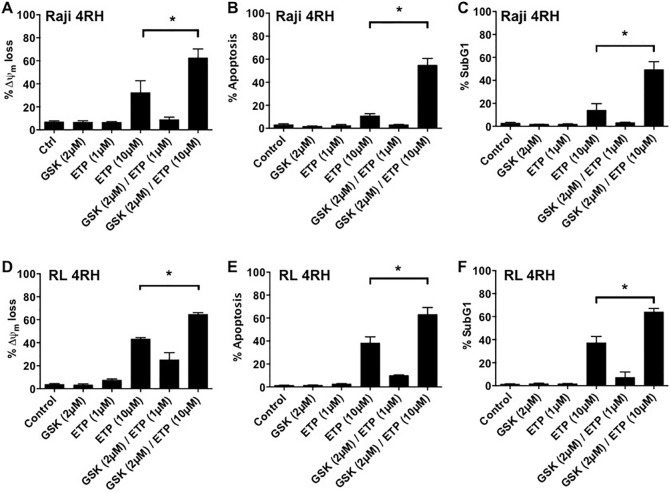
Raji 4RH and RL 4RH cell lines were treated as indicated for 14 days **A-F.** Apoptosis was assessed by flow cytometry analysis of TMRE, surface exposure of phosphotidylserine (annexin V staining) coupled with propidium iodide uptake and cell cycle analysis (subG1 analysis). All experiments represent mean ±SEM of 3 independent experiments. *p<0.05.

As shown in figures 1 and 2, etoposide treatment of RL-4RH and Raji-4RH cells is ineffective at inducing apoptosis, though is able to mediate cell cycle arrest and an increase in cellular senescence. Further, Figure 4 demonstrates the potential of combination treatment to effectively promote p53 expression and signaling activity, with concurrent loss of EZH2 and MDMX expression. While p21 and senescence were still significantly promoted by combination, determination of overall apoptotic response to combination treatment compared to single treatments was warranted. We assessed apoptotic response in RL- 4RH and Raji-4RH cells following 14-day exposure to single and combination treatments (Figure 5). Of the investigated treatment groups, single etoposide exposure at 10μM was sufficient to induce modest increases in hallmarks of apoptosis in both cell lines, including loss of mitochondria potential, increased cell surface exposure of phosphotidylserine, and DNA fragmentation (Figure 5A-5F). Excitingly, combination treatment involving a non-cytotoxic concentration of GSK126 (2μM) with 10μM etoposide resulted in significant overall increases for all readouts of apoptosis in both cell lines (Figure 5A-5F). These results validated that EZH2 is a major determining effector of the cellular response to therapy induced p53-DNA damage.

### Apoptosis induced by combination treatment is p53 dependent

**Figure 6:**
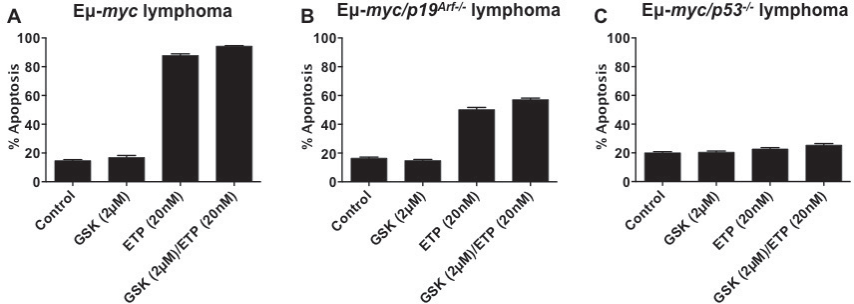
Eμ-*myc*, E-*myc/p19^Arf−/−^*, and Eμ-*myc/p53^−/−^* murine B-cell lymphomas were treated as indicated for 72 hours **A-C.** Apoptosis was assessed by flow cytometry analysis of surface exposure of phosphotidylserine (annexin V staining) coupled with propidium iodide uptake. Results represent mean ±SEM of 3 independent experiments.

It was previously reported that EZH2 inhibition directed response to DNA damage towards an apoptosis that was independent of p53 [7]. To investigate this, we utilized Eμ-*myc* B-cell lymphoma cell lines [15]. As expected, figure 6A shows Eμ-*myc* lymphomas exposed over 3 days were highly sensitive to etoposide-induced apoptosis, both as a single agent and in combination with GSK126. GSK126 single agent activity had minimal effect with inducing apoptosis in Eμ-*myc* lymphomas (Figure 6A). Eμ-*myc* lymphomas devoid of *p19^Arf^ (*Eμ-*myc/p19^Arf−/−^*) retain wild type p53, though its expression is dampened due increased p53 proteasome degradation by ubiquitin ligase activity [16]. With this, etoposide and combination were significantly effective with inducing apoptosis in Eμ-*myc/p19^Arf−/−^* lymphomas following 3-day drug exposure (Figure 6B). Deletion of p53 in Eμ-*myc/p53^−/−^* lymphomas resulted in complete abrogation of etoposide and combination induced apoptosis. These data indicate that apoptosis induction by combination of GSK126 and etopooside is dependent on p53 activity.

## DISCUSSION

Etoposide represents one of multiple genotoxic chemotherapies used in conjunction with rituximab to treat aggressive B-cell lymphomas. Unfortunately, upon resistance to these treatments, no current therapeutic strategies are available, highlighting a critical need to identify novel treatment options. Resistance to therapy often involves attenuation or inhibition of the apoptotic response by the tumor cell. Mechanisms involve over-expression of anti-apoptotic proteins like BCL-X_L_ and loss of expression/function of the tumor suppressor protein p53. Therapy restoration of p53 activity is an intriguing approach, and recent success from clinical trials has renewed enthusiasm for p53-based cancer therapy, via targeting of MDM2-p53 and MDMX-p53 interfaces. Adding complexity to the restoration of p53 activity, is achieving the desired cellular response by p53 to mediate apoptosis in response to DNA damage. P53 action in response to DNA damage most often occurs by the activation of genes involved in cell-cycle arrest, senescence, and apoptosis. Of interest, multiple studies indicate that p53-mediated senescence can impair the apoptotic response to chemotherapy in various cancers [3-6], resulting in chemo-resistance and limited patient response to such therapies.

It has been previously shown that the histone methyltransferase, EZH2 is a critical regulator of cell fate in response to DNA damage. Inhibition of EZH2 has resulted in cell apoptosis rather than cell cycle arrest in response to DNA damage induced by genotoxic drugs including etoposide [7]. Our data coincides with these results, as we show that single etoposide treatment of drug resistant B-cell lymphoma cell lines induces DNA damage associated with cell cycle arrest and senescence. Additionally, EZH2 inhibition by GSK126 combined with etoposide treatment altered the cell response to apoptosis, supporting the mechanistic role of EZH2 determining cell fate in response to DNA damage.

An important p53 target gene is the cyclin dependent kinase inhibitor, p21, which governs initial cell cycle arrest in cells undergoing senescence or apoptosis [13]. In addition, p21 is proposed to negatively regulate p53- mediated apoptosis [13]. Our results indicate a marked p21 response following short-term etoposide treatment as expected. However, long-term analysis following exposure to etoposide or GSK126 resulted in sustained p53-p21 expression by single etoposide and combination treatments. As, stated, maintained expression of the p53-p21 axis is well associated with DNA damage induced cell cycle arrest and senescence by activation of the Rb- E2F1 pathway [13]. This occurs concurrently with the inability of p53 to initiate its apoptotic-signaling pathway. Our data show that both etoposide single and combination treatment result in maintained p53-p21 expression with associated senescence. However, combination treatment significantly increased the apoptotic response in both RL- and Raji-4RH cell lines. This data suggests that indeed EZH2 significantly governs the cellular response to DNA damage. Pro-apoptotic targets of p53 include the BH3 only proteins, PUMA and NOXA. Expression analyses of these two target genes showed no significant alterations following any treatment (data not shown), indicating that the ability of EZH2 inhibition to alter the cellular response to etoposide mediated DNA damage was via alternate mechanisms. Wu et al. described that either FBXO32 mediated p21 proteasome degradation or CHK1 inhibition explained underlying EZH2 regulation of cell fate decision in response to DNA damage [7]. Our results did not investigate relative expression of FBXO32, though as mentioned above, our experiments following long-term exposure to etoposide and combination did show that p21 expression was maintained. The difference in p21 response observed by this study and Wu et al could be context dependent, as different cancer models were investigated.

Previously, it had been shown that RL- and Raji- 4RH multi-drug resistant cell lines displayed aberrations involving the intrinsic apoptotic pathway [17]. Resistant lines compared to their parental lines displayed loss of the BCL-2 pro-apoptotic proteins BAK and BAX as well as gain of the anti-apoptotic protein BCL-X_L_ [17]. All three proteins are known to act on mitochondria integrity during apoptosis. Further, our data indicates that mitochondrial damage is significantly increased by combination of etoposide and GSK126. Because EZH2 acts to silence gene expression via H3K27me3, we evaluated the response of BAK and BAX following all treatments, though neither target was elevated (data not shown), indicating that the loss of expression of these genes is independent of EZH2. Protection from mitochondria damage by BCL- 2 and BCL-X_L_ has been previously demonstrated to induce growth arrest or senescence while inhibiting apoptosis following chemotherapy. A previous study by Hayward et al [18] also indicated that BCL-X_L_ inhibited p53-dependent apoptosis following treatment with the topoisomerase I inhibitor irinotecan. More recently, increased expression of BCL-X_L_ expression mediated therapy resistance in B-cell lymphoid malignancies. BCL-X_L_ expression was regulated through a microRNA (miR) epigenetic mechanism. Apoptotic response was restored following de-repression of miR-377 with concurrent loss of BCL-X_L_ expression [19]. Our data also indicate that loss of BCL-X_L_ protein expression associated with increased apoptotic response to combination treatment, adding that EZH2 potentially regulates BCL-X_L_ via epigenetic mechanisms.

Most strikingly, we observed a significant increase of MDMX protein expression in RL- and Raji-4RH cell lines compared to their respective parental lines. MDMX is a part of the MDM family that function as critical inhibitors of p53 expression and function [20]. Upon DNA damage, MDMX protein stability is regulated by MDM2, which induces MDMX proteasome degradation [20]. In our experiments, etoposide single treatment induces loss of MDMX protein expression, which was further enhanced by combination treatment. This data also indicates that the regulation of MDMX protein involves epigenetic regulation down stream of DNA damage.

Because of the toxic side effects that accompany chemotherapy, utility of a combination therapy approach that directs DNA damage response towards apoptosis and allows for lower chemotherapy dosing would provide great benefit to patients. Overall, we demonstrate that inhibition of histone methyltranferase EZH2 regulates cell fate decisions in response to genotoxic chemotherapy in multi-drug resistant models of B-cell lymphoma. Further, this data suggests novel information that BCL-X_L_ and MDMX, important regulators of apoptotic and p53 signaling respectively, are in part epigenetically regulated by EZH2. Ultimately, these results provide exciting potential for the combination of genotoxic therapy with inhibitors of EZH2 as a novel therapeutic strategy for patients with aggressive treatment resistant B-cell lymphomas.

## MATERIALS AND METHODS

Eμ-*myc*, Eμ-*myc*/*p53*^−/−^, and Eμ-*myc/p19^Arf−/−^* B-cell lymphomas were kindly provided by Dr. Ricky Johnstone. Eμ-*myc* lymphomas were cultured in 6-well plates in high-glucose DMEM (Dulbecco modified Eagle medium) supplemented with 10% fetal calf serum, penicillin/streptomycin, 0.1 mM L-asparagine and 50 μM 2-mercaptoethanol in a 37°C, 10% CO2 humidified incubator. Human B-cell lymphoma cell lines (RAJI-4RH and RL-4RH) were kindly provided by Dr. Francisco Hernandez-Ilizaliturri and cultured under standard conditions (37°C, with 5% CO2) in RPMI (Roswell Park Memorial Institute) 1640 medium supplemented with 10% FBS and penicillin/streptomycin. Etoposide (Sigma-Aldrich) and GSK126 (Xcess Biosciences Inc.) were maintained in DMSO at 1mM and 10mM stock concentrations respectively.

### *In vitro* cell death and proliferation assays

Cells were seeded in 24-well plates to a final concentration of 1.5×10^4^ cells/well. The day after, the selected compounds were added at the indicated concentrations. Viability tests were performed by propidium iodide (PI) uptake (Sigma Aldich), Annexin V staining (BD Pharmigen), cell-cycle analysis or Tetramethylrhodamine ethyl ester (TMRE) staining (Sigma Aldrich) according to the manufacturer “s instructions. Proliferation assays were performed using the MTS reagent (Promega) prepared according to manufacturer's instructions. Senescence was determined using a senescence detection kit as per manufactures instructions (Biovisions Incorporated).

### Quantitative real-time PCR

RNA isolation from cell lines was performed by TRIzol extraction. Synthesis of cDNA was performed according to iScript cDNA Synthesis Kit (Bio-Rad Laboratories, Hercules, USA). 1μg of RNA was added to a master mix containing nuclease free H_2_O, and reagents (5X iScript reaction mix + iScript reverse transcriptase) from the kit in a total volume of 20μl. cDNA was diluted 1:4 prior to qRT-PCR. PCR primers were designed with NCBI's primer blast tool (http://www.ncbi.nlm.nih.gov/tools/primer-blast/), with a melting temperature 57-63°C and a resulting product size of 75-200bp. Primers were obtained from Integrated DNA technologies (Coralville, USA). Primer sequences were *MDMX* (F: TGGAAGGACGGGCCATCT, R: TGCTATAAAAACCTT AATAACCAGCTGAA), *TP53* (F: CACATGACGGAGGTTGTG, R: ACACGCAAATTTCCTTCCAC), *P21* (F: GACCTGTCACTGTCTTGTA, R: CCTCTTGGAGAAGATCAGCCG) and *GAPDH* (F: GTCTTCACCACCATGGAGAAG, R: CAAAGTTGTCATGGATGACCTTGG). Each PCR reaction was carried out in technical triplicates in a 10μl volume utilizing SYBR Green Master Mix (Bio- Rad Laboratories, Hercules, USA). GAPDH was used a control gene. The resulting Ct-values for each gene were normalized to the expression values of GAPDH.

### Western blotting and antibodies

*Whole Cell Lysate Preparation (WCL)*: Cells were harvested and lysed with RIPA buffer (Sigma Aldrich, USA) + 1X P-STOP + 1X PIC (Roche) for 30 minutes on ice. Eppendorf tubes were vortexed every ten minutes for 10 seconds. After cell lysis tubes were centrifuged at 13,000 rpm for 15 minutes at 4°C. Supernatant of each tube was collected and transferred to a new tube.

*Histone Extraction*: Histone extractions were performed using the Epigentek (EpiQuik Total Histone extraction kit OP-0006) histone extraction kit.

Protein concentrations of whole cell lysates (WCL) and histone extractions were measured by the bradford protein assay (Bio-Rad laboratories). Protein lysates (50μg WCL, 5μg Histone Extraction) where separation using 4-15% by SDS-PAGE gels (Bio-Rad). The proteins were transferred from the SDS-PAGE gel onto nitrocellulose membrane (0.2 μm) (Bio-Rad, Hercules, CA) via the semi-dry method (Bio-Rad, Hercules, CA) for 35 minutes at 15V. Membranes were blocked in either 5% skim milk or BSA in 0.1% tween-PBS (tPBS) for 1-hour at RT. Membranes were washed briefly 3x with tPBS prior to primary antibody incubation at 4°C over night. Membranes were then washed 3×10 minutes before the addition of secondary horseradish peroxidase (HRP)-conjugated antibodies (Bio-Rad, Hercules, CA) diluted in tPBS. After incubation at RT for 1-hour with agitation the membranes were washed 3×10 minutes in tPBS. The immunoreactive bands were visualized by enhanced chemiluminescence with ECL detection reagents (GE Healthcare Life Sciences, UK). The blots were exposed to Bio film for 1 second-10 minutes. The films were then developed in a Kodak film developer. To estimate molecular weight of bands a pre-stained protein ladder was used (Bio-Rad, Hercules, CA). The following primary antibodies were used for Western blotting: Tp53 (Cell Signaling), MDMX (ProteinTech), EZH2 (Cell Signaling), H3K27me3 (Cell Signaling), Total Histone H3 (Cell Signaling), GAPDH (Cell Signaling). Each western blot data has been confirmed in at least two biological independent experiments.
